# Synergistic cryoablation and immunotherapy in the treatment of metastatic melanoma of unknown primary

**DOI:** 10.1016/j.jdcr.2026.07.055

**Published:** 2026-08-04

**Authors:** Anam Adil, Wenhua Liu, Adam Levy, Aleksandar Krunic

**Affiliations:** a Rush University Medical College, Chicago, Illinois; b Department of Dermatology, University of Illinois Chicago, Chicago, Illinois; c Department of Otolaryngology, Advocate Illinois Masonic Medical Center, Chicago, Illinois; d Department of Dermatology, Northwestern University, Chicago, Illinois

**Keywords:** cryoablation, cryotherapy, ear auricle, immune checkpoint inhibitors, melanoma, neoplasms, unknown primary

## Introduction

Melanoma of unknown primary (MUP) presents as a rare metastatic melanoma with no identifiable primary lesion, complicating both diagnosis and management. We report a case of MUP of the left concha initially treated with radical auriculectomy. Subsequent presentation of metastatic melanoma on the stump was successfully managed with 1 year of pembrolizumab and aggressive contact cryosurgery to the metastatic lesions.

## Case report

**Fig 1:**
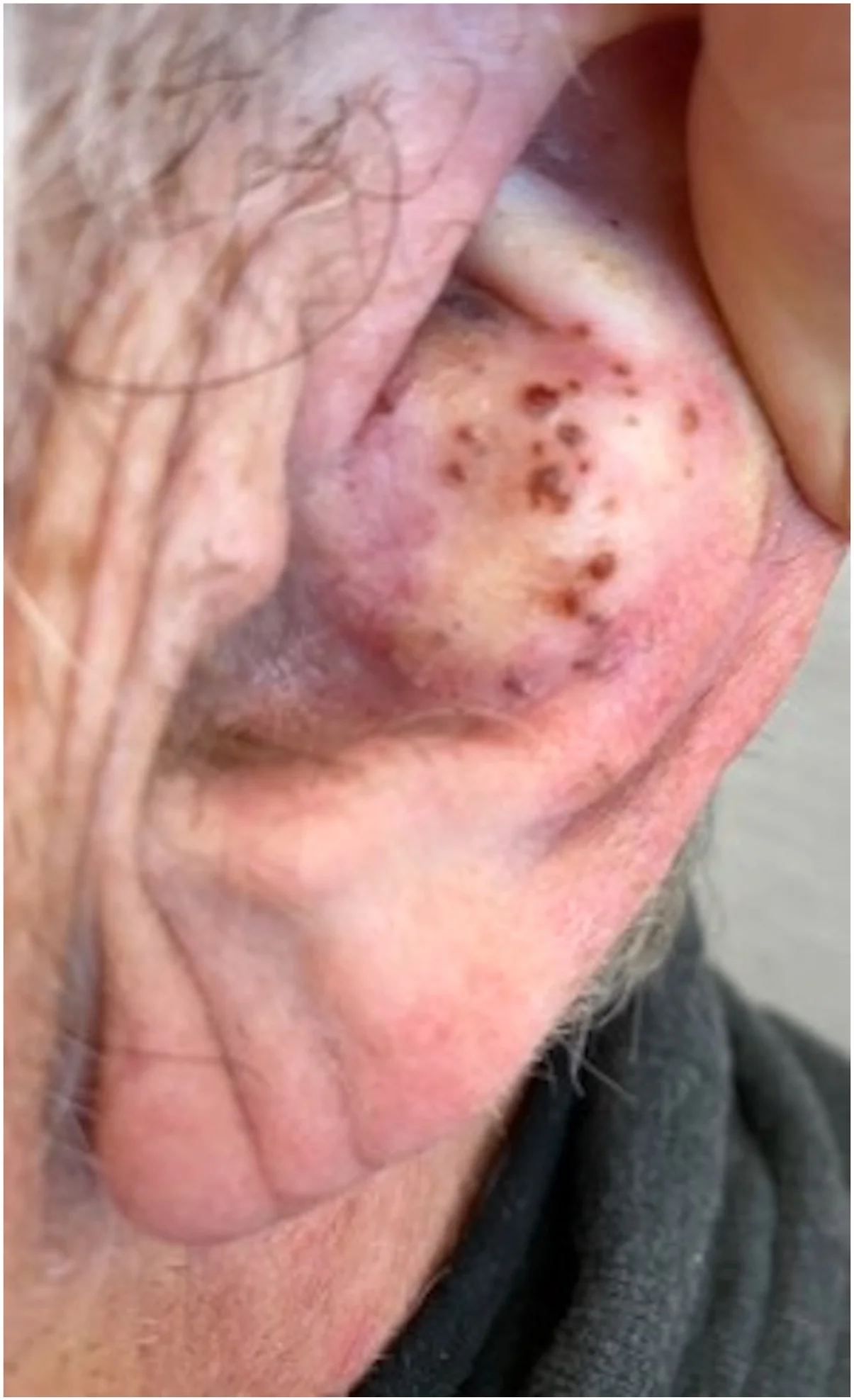
Cluster of 2-3 mm pigmented papules involving the left scapha and concha.

**Fig 2:**
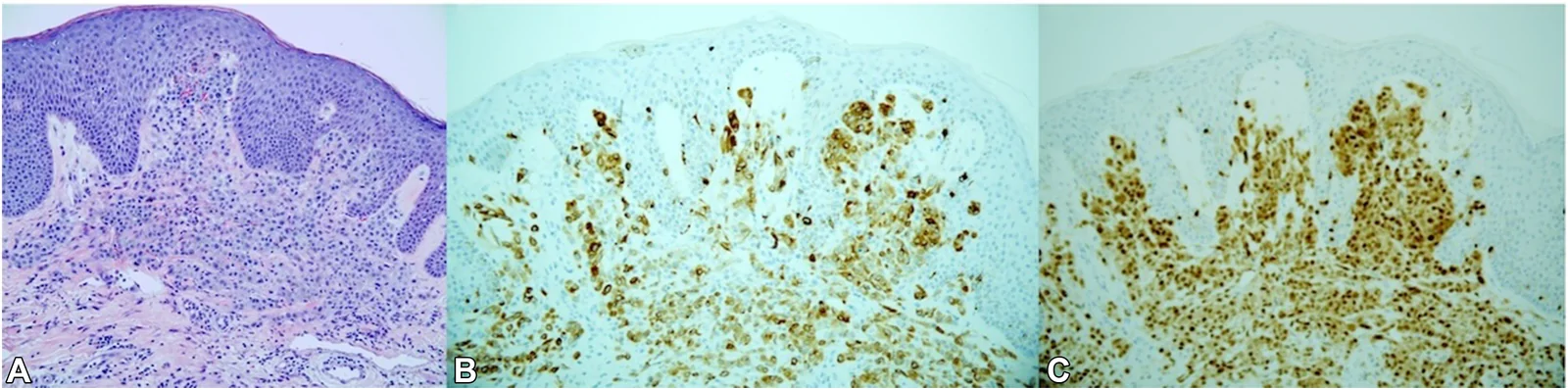
**A-C,** Punch excision of the left conchal lesion demonstrates **(A)** Nodular dermal proliferation of atypical melanocytes arranged in nests without an epidermal connection or in situ component (hematoxylin-eosin stain, ×200) **(B)** Atypical melanocytes are positive for Melan A (×200) **(C)** MITF positivity in tumor cells (×200). *MITF.* Microphtalmia-associated transcription factor.

A 73-year-old male presented with a cluster of asymptomatic, 2-3 mm, brownish-black papules, involving the left scapha and concha, present for 4 months (Fig 1). He was otherwise asymptomatic and had an unremarkable medical history. A complete dermatologic examination of the skin, mucosal surfaces, and adnexa, as well as otolaryngologic and ophthalmologic evaluations, revealed no primary or regressed melanocytic lesions. Detailed otologic exam further revealed an intact external auditory canal and unaffected middle and inner ear structures. A punch excision of a papule on the superior aspect of the scapha was performed, revealing a Melan A-positive cluster of atypical melanocytes situated in the dermis, without epidermal connection or an *in situ* component, consistent with metastatic melanoma (Fig 2, *A* and *B*). The tumor was microphtalmia-associated transcription factor-positive and BRAF-mutation-negative (Fig 2, *C*). Imaging studies, including computed tomography scan, positron emission tomography-computed tomography, and sentinel node evaluation, did not disclose any primary or metastatic tumors. Consequently, the patient was diagnosed with metastatic MUP. Additionally, LDH levels were not elevated, classifying the patient in the stage IV1a (0).

**Fig 3:**
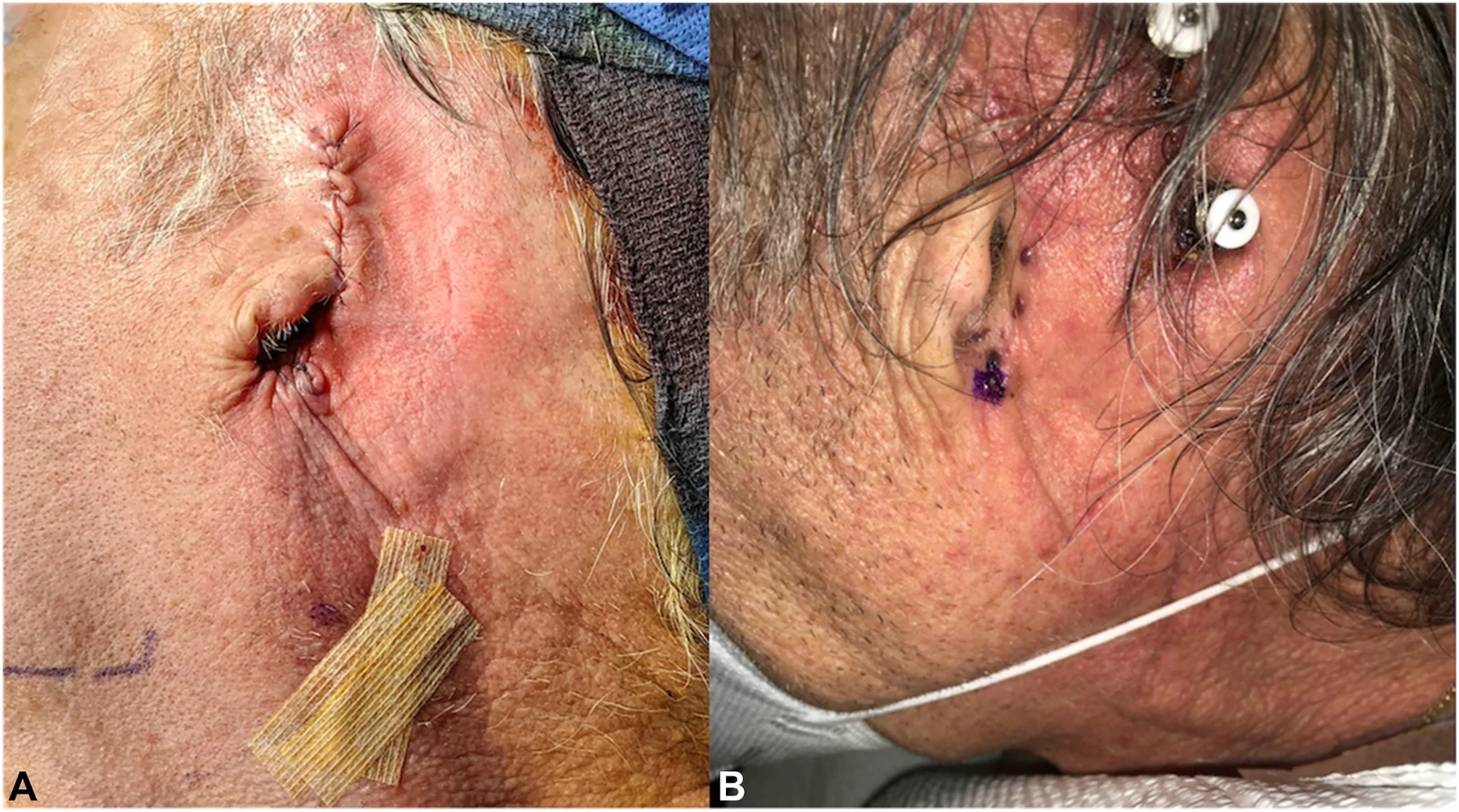
**A** and **B, (A)** Unremarkable scar following radical auriculectomy of the left external ear **(B)** Recurrence of melanoma metastases presenting as brownish-black papules on the amputation stump along the opening of the left external auditory canal.

One month later, the patient underwent radical auriculectomy with complete removal of the external ear (Fig 3, *A*). The postoperative course was unremarkable, and the following month, he was started on adjuvant therapy with pembrolizumab 200 mg IV every 3 weeks for a total of 17 cycles (12 months).

Three months postoperatively, three 3-5 mm pigmented papules appeared on the amputation’s stump along the external auricular canal (Fig 3, *B*). Subsequent biopsy again revealed clusters of atypical melanocytes in the dermis with no epidermal connection, consistent with recurrent melanoma. At this stage, the patient refused more extensive surgery and mutilation of the left side of the face and head, preferring more conservative treatment. He was therefore considered inoperable.

A cryosurgical approach was then considered. All 3 metastatic nodules were treated by cryosurgery, using a 6 mm cryoprobe (M-559702-4662, Brymill Cryogenic Systems). Cryosurgery was performed in 2 cycles, each with 60 seconds of freezing time, and a 5 mm halo around the treated lesions. After the second cryosurgery session, all nodules regressed. Pembrolizumab therapy was completed the following year, and the post-treatment course was unremarkable.

**Fig 4:**
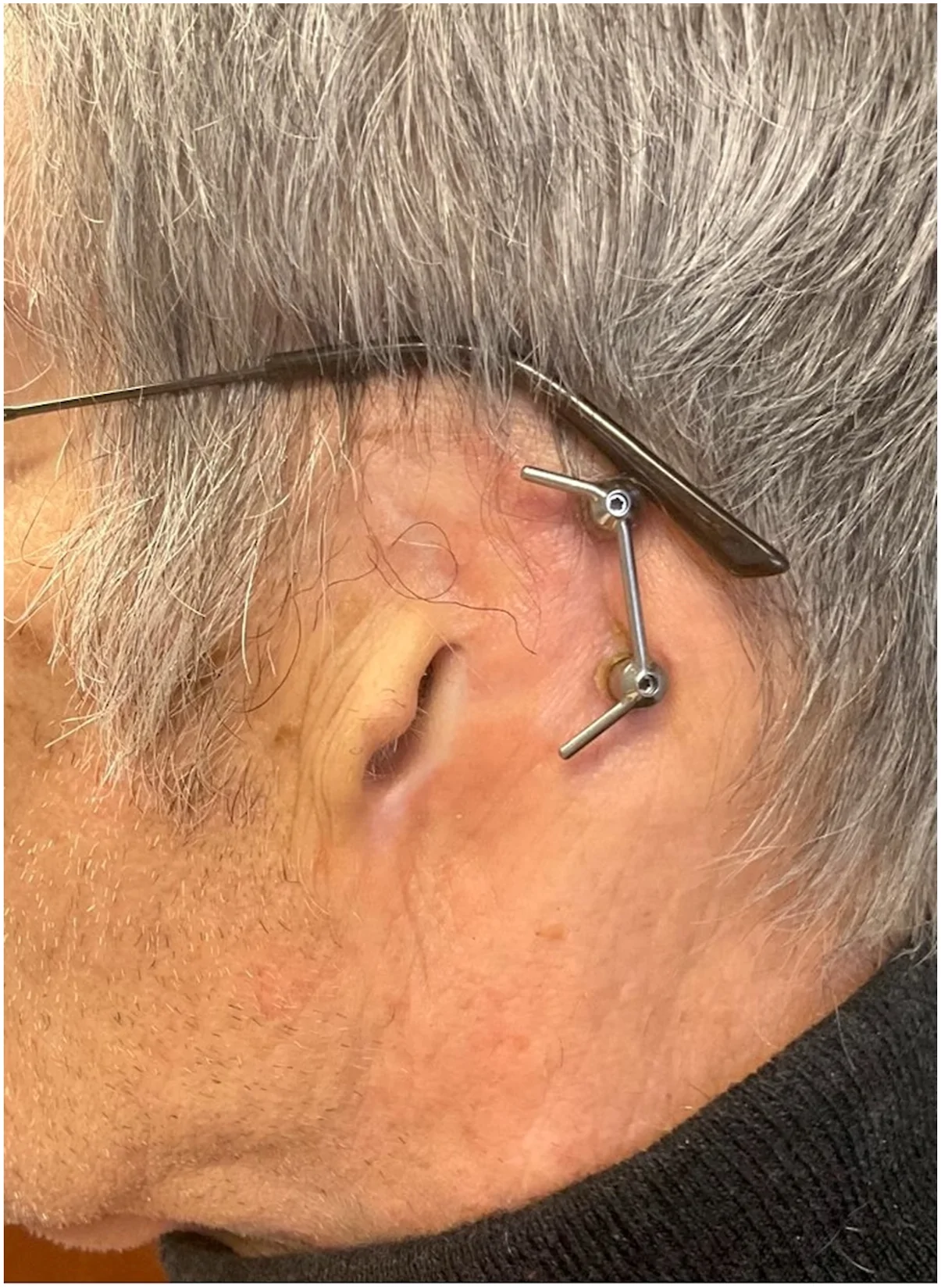
No evidence of recurrent disease at 3-year follow-up.

Three-year follow-up revealed no residual or recurrent disease. The patient remains in good health, with 2 subsequent annual imaging studies negative for disseminated disease (Fig 4).

## Discussion

Melanoma of the external ear accounts for approximately 7% to 20% of head and neck melanomas and about 4% of all skin melanomas.^1^ Most tumors in the auricular region are primary melanomas, typically of the superficial spreading malignant melanoma or lentigo maligna melanoma types. MUP constitutes 3% to 4% of all melanomas and is extremely rare in the auricular region.^2^^,^^3^

Histopathologic interpretation of MUP usually pertains to dermal and/or subcutaneous foci of atypical melanocytes with minimal perifocal inflammation in the absence of junctional activity or without any connection with the epidermis. Diagnosing MUP requires a thorough examination for possible regressed primary lesions or other primary tumor sources, which can be challenging to detect. In addition, due to its low incidence, establishing optimal treatment strategies remains difficult.

While surgery is typically the primary treatment for localized melanoma, further excision was not feasible in our case due to the anatomic location and risk of significant mutilation.

Therefore, we pursued a combination of local cryosurgery and systemic immunotherapy with pembrolizumab, aiming to achieve local control while preserving function and quality of life.

Notably, the external auditory canal, middle ear, and inner ear were unaffected, placing our patient in a more favorable category. Involvement or primary origin of melanoma in the external auditory canal typically leads to extensive metastatic disease in the temporal bone, parotid gland, and superior cervical lymph nodes.^4^

The combination of cryoablation with immunotherapy represents an emerging therapeutic strategy that leverages complementary mechanisms of action. Cryoablation can be used to reduce tumor burden in unresectable melanoma lesions with high metastatic load and may slow the rate of tumor spread in metastatic disease.^5^ Among various ablative techniques, cryoablation with cryoprobe application allows deeper penetration of the cryogen and more efficient cryonecrosis of malignant cells, approximately 1.3 times the peripheral freezing halo, while also inducing a systemic antitumor immune response.^6^ Cryonecrosis releases intact tumor antigens that are processed by antigen-presenting cells and presented to tumor-specific T cells.^6^^,^^7^ Technical parameters of cryoablation may further influence its immunologic effects, with rapid freeze rates producing greater tumor necrosis and stronger local and systemic immune responses than slower freeze rates.^8^ However, cryoablation alone may generate an immune response that is insufficient to overcome tumor-induced immune suppression. Immune checkpoint inhibitors, including cytotoxic T-lymphocyte-associated protien 4 and programmed cell death protein 1/programmed death-ligand 1 blockade, therefore augment this response by sustaining antitumor T-cell activity.^7^

Cryotherapy is particularly valuable for treating skin melanomas in anatomically challenging locations, such as the external ear, periorbital area, and nasal ala, where surgery is not feasible and can result in significant cosmetic and functional defects. This technique enables more precise removal of tumor tissue while sparing healthy surrounding tissue, potentially reducing surgical defects by two-to threefold compared to conventional excisional techniques with 1-2 cm margins.^9^ Despite these advantages, cryotherapy has limitations. Unlike conventional excision, cryotherapy does not provide histologic margin assessment. In addition, healing occurs by secondary intention and may be associated with ulceration, delayed wound healing, or infection.^6^ Furthermore, the efficacy of cryoimmunotherapy may also be influenced by cryoablation parameters, as well as interpatient differences in tumor immunogenicity and host immune responses, which together may affect the magnitude of the systemic antitumor immune response.^7^^,^^8^ Consequently, careful clinical surveillance remains essential to monitor local control and detect recurrence.

The favorable outcome in our patient, with sustained disease-free status after completion of therapy, suggests that combining local and systemic therapies may provide effective disease control in MUP when standard interventions are impractical. The clinical presentation in our patient suggests a probable primary lesion on the helical rim that may have regressed, resulting in cutaneous metastatic deposits, similar to the case described by Kolsidopulos and Skoulakis.^3^ However, despite detailed examination and imaging, no primary lesion was identified in our case.

Although current therapeutic approaches for MUP largely mirror those for advanced melanoma, the combination of cryosurgery and immunotherapy offers a promising, synergistic alternative for nonoperable cases and preservation of quality of life. Our experience highlights the potential value of this approach, particularly in challenging anatomic sites, and supports further investigation into its role in complex melanoma management.

## Conflicts of interest

None disclosed.
